# Cervical Radiculopathy and Vertebrobasilar Insufficiency Secondary to Intraneural Foramen Vertebral Artery Loop: A Case Report

**DOI:** 10.7759/cureus.64478

**Published:** 2024-07-13

**Authors:** Raghad Z Aljohani, Maryam Alshanqiti, Waleed R Murshid

**Affiliations:** 1 Neurosurgery, King Fahad General Hospital, Madinah, SAU; 2 Neurological Surgery, Prince Mohammad bin Abdulaziz Hospital, Ministry of National Guard-Health Affairs, Madinah, SAU

**Keywords:** tortuous vertebral artery, vertebrobasilar insufficiency, cervical radiculopathy, vascular loop, vertebral artery

## Abstract

This case report describes a 40-year-old male who presented with chronic neck pain radiating to the left upper limb, associated with weakness and numbness. He also had symptoms of vertebrobasilar insufficiency. Imaging revealed an intraneural foramen vertebral artery (VA) loop compressing the C3 nerve root. Conservative management was ineffective, prompting surgical decompression via a left C2-C3 facetectomy and foraminotomy. The patient experienced immediate pain relief and gradual improvement in weakness, with complete resolution of symptoms at the six-month follow-up. This case highlights the potential for VA loops to cause radiculopathy and the successful use of surgical decompression for treatment.

## Introduction

VA loops, also known as dolichoectasia, are an uncommon cause of cervical radiculopathies. The incidence of VA loop anomalies is approximately 3% [1]. Not all VA loops result in cervical radiculopathy. While 40% of patients with VA anomalies exhibit symptoms due to the presence of a VA loop, 60% of other VA loops are discovered incidentally, often during evaluations for traumatic spinal injuries, occipitocervical pain, or degenerative disc changes [2]. A VA loop, or tortuosity, can be a congenital or acquired anomaly [3]. Although the exact pathogenesis remains unclear, there are possible explanations and risk factors, including degenerative narrow disc space, that are hypothesized to cause relative elongation of the VA, potentially resulting in a VA loop, cervical traumatic events, damage to the VA from hypertension, and/or atherosclerosis [2]. Patients with VA loops may present with a range of symptoms, from numbness and weakness in the upper extremities to true occipital neuralgia [4]. The initial treatment approach is conservative management; however, if this is unsuccessful, surgical intervention may be necessary to decompress the affected nerve root, either directly or through arterial transposition.

## Case presentation

Patient information

A 40-year-old male, not known to have any medical comorbidities, had no past surgical history and was not on any medication. He presented to us complaining of occipital headache and chronic neck pain, which had been radiating to the left upper limb for one year. His pain was severe, electrical in nature, and disturbed his daily physical activity, exacerbated by driving and relieved by shoulder abduction. He also reported weakness and numbness in the same limb. The pain was associated with nausea and vomiting. His symptoms were not improved by conservative measures, including non-steroidal anti-inflammatory drugs (NSAIDs), gabapentin, baclofen, or a soft cervical collar. There was no history of cervical spine injury.

Clinical findings

He had mild weakness all over his left upper limb (+4/5) in comparison to his right (5/5). The weakness was more pronounced over the triceps (elbow extension, −4/5), and he also had weakness in the deltoid (shoulder abduction, +4/5). He was complaining of hypoesthesia all over his left arm, left ear, and left side of the neck (C2-C3 dermatome). 

Diagnostic assessment

**Figure 1:**
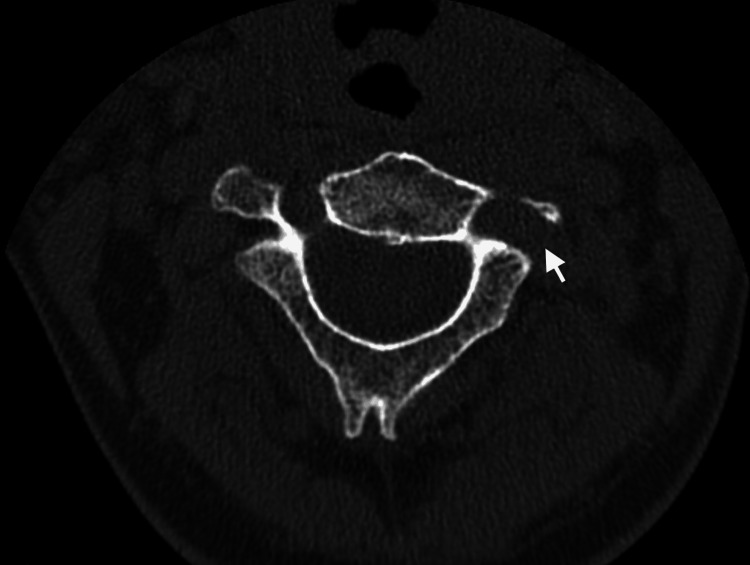
CT cervical spine showing the expanded C2 foramen transversarium

**Figure 2:**
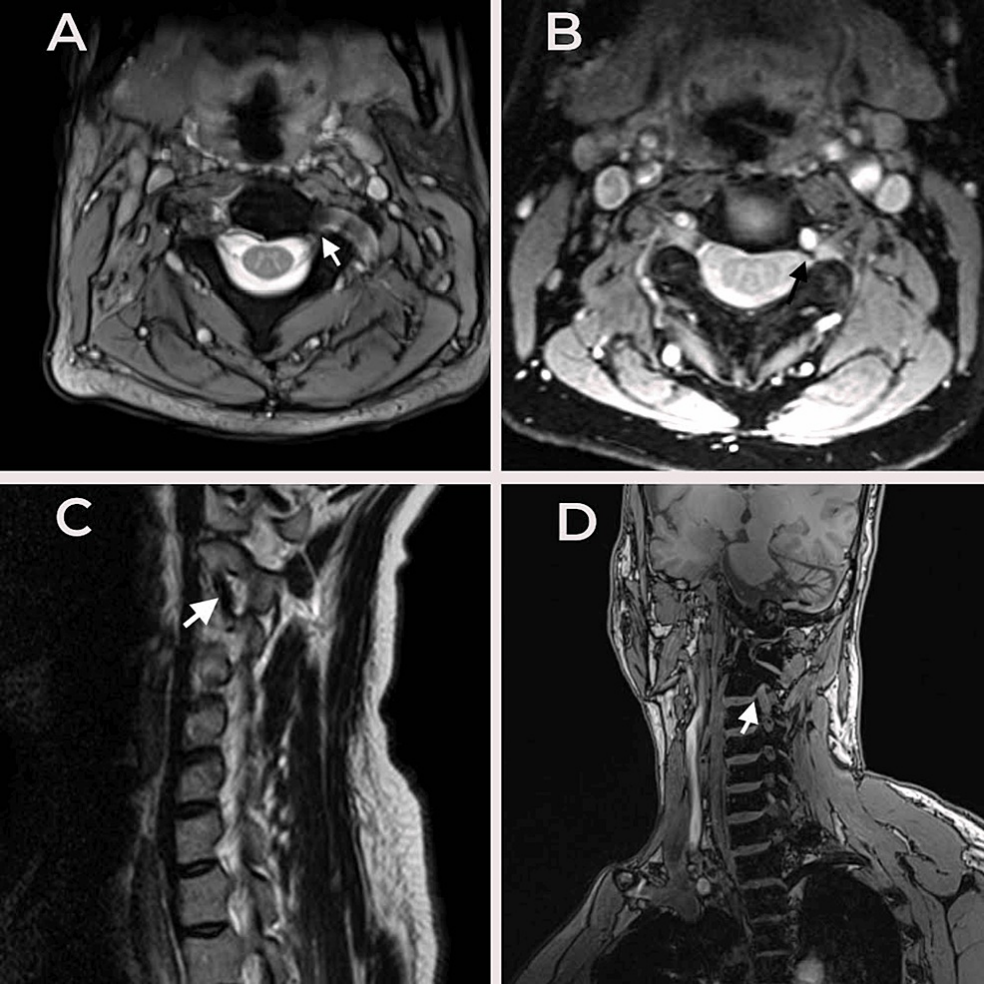
(A) T2 MERGE, (B) MRA, (C) T2 sagittal MRI, and (D) T1 VIBE MR The arrows point to the abnormal left vertebral artery course within the foramen, compressing the C3 root. MRA, magnetic resonance angiography; MRI, magnetic resonance imaging

He was evaluated with cervical CT and magnetic resonance imaging (MRI). The CT cervical spine revealed an expanded left C2 transverse foramen with a markedly thin medial wall (Figure 1). This was followed by MRI and magnetic resonance angiography (MRA) of the cervical spine, which showed a tortuous left V3 segment of the VA looping within the neural foramen and compressing the C3 nerve root (Figures 2A-2D).

Therapeutic intervention

The patient underwent a posterior approach, including a left C2-C3 facetectomy and a C2-C3 foraminotomy.

Follow-up and outcome

Postoperatively, the pain resolved immediately, and he had a gradual improvement in weakness, which resolved within days of the surgery. At the six-month follow-up, the patient was doing well, and his previous symptoms had resolved completely.

## Discussion

Vascular loop causing radiculopathy is a rare but noteworthy clinical entity. Benny et al. [5] highlighted its rarity in their literature review, identifying only five cases of cervical radiculopathy secondary to VA tortuosity up to that time. The incidence appears to be similar between males and females, with the C4-C5 level most commonly affected, typically unilaterally. A cadaveric study reported a 2.7% incidence of tortuous vertebral arteries, suggesting that while the condition is uncommon, it is not exceedingly rare [6].

The pathophysiology of VA loop formation remains unclear [6]. Sakaida et al. [7] proposed that the elongation of the VA is secondary to disc space narrowing, leading to bone remodeling and erosion through arterial pulsation, resulting in an enlarged neural foramen and subsequent nerve root compression. Oga et al. [8] supported this theory, finding a direct correlation between the grade of cervical spondylosis and the degree of VA tortuosity.

Diagnostic modalities play a crucial role in identifying this condition. X-rays typically reveal adjacent bone erosion and sclerotic margins. Computed tomography angiography (CTA) is particularly beneficial, showing nerve root compression by the hyperdense VA loop. MRI manifests as signal voids extending to the neural foramen. However, MRA or conventional angiography is considered the standard diagnostic tool, with MRA preferred due to the lower radiation risk [9]. 

Management strategies vary from conservative to surgical. Detwiler et al. [10] reported successful outcomes using vascular decompression in three patients, while Sakaida et al. [7] treated another patient similarly. Horgan et al. [11] managed patients conservatively, noting symptom improvement with blood pressure control and NSAIDs. Conservative management is often preferred in young patients to avoid surgical risks, though it may fail, necessitating surgical options such as microvascular decompression and foraminotomy [10].

The literature reports mixed outcomes for conservative versus surgical management. Paksoy et al. [12] found that conservative management, including physiotherapy, antihypertensives, anxiolytics, and antidepressants, was effective in most patients. However, six patients who did not initially respond to conservative management ultimately improved over a 12-month follow-up period without surgery. Tonsbeek et al. [2] summarized the previously reported surgical management of cervical radiculopathy caused by VA vascular loop. The anterolateral approaches included reconstruction of the artery with end-to-end anastomosis, transposition of the artery with a sling, and loop mobilization with a Teflon plug. Posterior approaches, like in our case, included laminectomy with facetectomy and separation of the artery from the root. As a last resort, endovascular coil occlusion and vessel sacrifice for failed previous surgeries with persistent symptoms.

A case series by Hardmeier et al. [13] highlighted VA dissection as another cause of radiculopathy, with a noted correlation between VA tortuosity and cervical spondylosis. The dissection typically causes motor symptoms due to the anatomical proximity of the artery to the motor side of the nerve root, with C5 being the most commonly affected level.

Several other case reports illustrate diverse presentations and management approaches. For instance, Zimmerman and Farrell [14] reported cases where surgical intervention was necessary due to severe symptoms unresponsive to conservative measures. Similarly, Wood et al. and Khansuheb et al. [1,4] described surgical decompression and vascular mobilization techniques that resulted in symptom resolution. Sakadia et al. [7] reported a case similar in presentation to our patient, but they performed an anterior approach in contrast to our case, where we used a posterior approach. A 62-year-old man had been suffering from worsening radiculopathy on the left side at the C5 nerve root and bouts of dizziness for four months. Diagnostic imaging revealed that a VA had formed a loop and moved into the enlarged intervertebral space between the fourth and fifth cervical vertebrae (C4-C5). To address this, the patient had surgery to reconstruct the VA loop. This procedure involved only slight manipulation of the C5 nerve root and was performed using a left-sided anterolateral approach following a successful balloon occlusion test. After the surgery, the patient’s symptoms were relieved instantly, and he remained free of symptoms over the course of a two-year monitoring period. The favorable result from this case reinforces the view that surgical reconstruction is a preferable treatment for relieving pressure from a twisted VA, as it poses a smaller risk of damaging the nerve root and also leads to better blood flow in the posterior circulation.

## Conclusions

In summary, VA loops causing radiculopathy are rare and can be challenging to diagnose and manage. The choice between conservative and surgical management should be individualized, considering the patient’s age, symptom severity, and risk of surgical complications. While conservative management may suffice for some, surgical intervention remains a viable option for those with persistent or severe symptoms. The evolving understanding of this condition underscores the importance of thorough diagnostic evaluation and tailored treatment strategies. Further studies are needed to establish standardized guidelines for managing this rare but significant clinical condition.
